# The effect of physical interventions on pain control after orthodontic treatment: A systematic review and network meta-analysis

**DOI:** 10.1371/journal.pone.0297783

**Published:** 2024-02-22

**Authors:** Junxiong Li, Siyu Li, Hongjun Chen, Jingzhe Feng, Ya Qiu, Lihua Li

**Affiliations:** 1 Department of Stomatology, Affiliated Hospital of North Sichuan Medical College, Nanchong, Sichuan, China; 2 Medical Research Center, Affiliated Hospital of North Sichuan Medical College, Nanchong, Sichuan, China; Hamadan University of Medical Sciences, ISLAMIC REPUBLIC OF IRAN

## Abstract

**Objective:**

Pain is a frequent adverse reaction during orthodontic treatment, which can significantly reduce treatment compliance and compromise the expected treatment effect. Physical interventions have been used to alleviate pain after orthodontic treatment, but their effectiveness is controversial. This study used a network meta-analysis to assess the efficacy of various physical interventions typically used in managing pain after orthodontic treatment, with a view to provide evidence-based recommendations for representative interventions for orthodontic pain relief during peak pain intensity.

**Methods:**

A systematic search of six electronic databases, from their respective inception dates, was conducted to identify relevant literature on the efficacy of various typical physical interventions for managing pain after orthodontic treatment. Literature screening was performed according to the Cochrane System Evaluator’s Manual. Stata 16.0 was used to assess heterogeneity, inconsistency, publication bias, and sensitivity to generate an evidence network diagram and conduct a network meta-analysis.

**Results:**

In total, 771 articles were reviewed to collect literature on interventions, including low-level laser therapy (LLLT), vibration, acupuncture, and chewing. Of these, 28 studies using a visual analog scale (VAS) as an outcome indicator were included. The results showed that LLLT, vibration, acupuncture, and chewing effectively relieved the pain symptoms in patients after orthodontic treatment. At 24 h post-treatment, LLLT (surface under the cumulative ranking curve [SUCRA] = 80.8) and vibration (SUCRA = 71.1) were the most effective interventions. After 48 h of treatment, acupuncture (SUCRA = 89.6) showed a definite advantage as the best intervention.

**Conclusion:**

LLLT, vibration, acupuncture, and chewing can alleviate pain associated with orthodontic treatment. Among these interventions, acupuncture was found to be the most effective at 48 h after orthodontic treatment. In addition, acupuncture demonstrated long-lasting and stable pain-relieving effects. However, further studies are needed to determine the most suitable equipment-specific parameters for acupuncture in relieving pain associated with orthodontic treatment.

## Introduction

The average duration of orthodontic treatment is approximately 24 months [1], which implies the importance of patient compliance for effective treatment [2]. Pain is a frequent adverse reaction during orthodontic treatment, with an incidence rate of 72–100% [3]. It is an unpleasant sensory and emotional experience associated with or resembling actual or potential tissue damage [4]. Generalized orthodontic pain refers to any pain sensation caused by orthodontic appliances, such as mucosal ulcers, tongue discomfort, or gum damage. It can be experienced as discomfort, sensitivity, and pain in the teeth, which is typically attributed to tooth movement [5]. Orthodontic pain not only reduces quality of life but also affects pronunciation. The pain may lead to negative emotions that can cause short-term memory loss and learning disabilities [6]. Patients with dental anxiety may experience more severe pain after orthodontic treatment, which can significantly reduce treatment compliance and compromise the expected treatment effect [7]. The pain may even cause some patients to discontinue treatment prematurely because of their inability to tolerate the discomfort [8]. This is particularly true for removable mobility appliances. The most intense pain experienced after orthodontic treatment occurs within the first 24–48 h [9].

Pain typically motivates individuals to seek means of alleviation. However, conventional methods cannot alleviate pain in patients undergoing fixed orthodontic treatment. The use of physical interventions, such as low-level laser therapy (LLLT), vibration therapy, chewing therapy, and acupuncture, to alleviate pain after orthodontic treatment has been a topic of significant research in recent years. However, their effectiveness is controversial due to differences in experimental design, equipment parameters, and other factors among studies, leading to variable results.

To address this issue, we have conducted a network meta-analysis to evaluate the efficacy of these physical interventions typically used for pain reduction after orthodontic treatment.

## Methods

This study followed the Preferred Reporting Items for Systematic Reviews and Meta-Analyses-Network Meta-Analysis (PRISMA-NMA) guidelines (S1 Table) and was registered at PROSPERO (https://www.crd.york.ac.uk/prospero/; CRD42022304883).

### Search strategy

We conducted a thorough online search for eligible studies in five electronic databases: China National Knowledge Infrastructure, WangFang Database, Chinese Biomedical Literature Service System (SinoMed), PubMed, and Cochrane Library, from their respective inception dates up to May 31, 2023. We limited publications to those in the English and Chinese languages. The MeSH terms used in this study included orthodontics, pain, LLLT, vibration, chewing, acupuncture, transcutaneous electrical nerve stimulation (TENS), transcutaneous electrical acupoint stimulation (TEAS), and visual analog scale (VAS). The PubMed search strategies are shown in Table 1, as a representative example. The search items were appropriately adjusted to meet the requirements of each database in order to ensure the basic logical integrity of the search.

**Table 1:** Strategies used for searching PubMed for eligible publications.

Steps	Search
#1	"orthodontal"[All Fields] OR "orthodontic"[All Fields] OR "orthodontical"[All Fields] OR "orthodontically"[All Fields] OR "orthodontics"[MeSH Terms] OR "orthodontics"[All Fields]
#2	"pain"[MeSH Terms] OR "pain"[All Fields]
#3	"low-level light therapy"[MeSH Terms] OR ("low-level"[All Fields] AND "light"[All Fields] AND "therapy"[All Fields]) OR "low-level light therapy"[All Fields] OR ("low"[All Fields] AND "level"[All Fields] AND "laser"[All Fields] AND "therapy"[All Fields]) OR "low level laser therapy"[All Fields]
#4	"visual analogue scale"[All Fields] OR "visual analog scale"[MeSH Terms] OR ("visual"[All Fields] AND "analog"[All Fields] AND "scale"[All Fields]) OR "visual analog scale"[All Fields]
#5	#1 AND #2 AND #3 AND #4

### Inclusion criteria

We included publications on randomized controlled trials (RCTs) in which patients received their first orthodontic appliances (regardless of appliance brand, archwire model, applied force, amount of movement, and wearing frequency). The experimental group adopted LLLT, vibration, chewing (e.g., chewing gum), or acupuncture as treatment. Acupuncture included classical acupuncture, TENS, and TEAS, while the control group underwent routine treatment without any special intervention. Outcome indicators included VAS scores at 24–48 h after initiating orthodontic treatment.

### Exclusion criteria

We excluded non-RCT publications, as empirical evidence indicated that intervention effects in orthodontic research appear to be inflated in non-RCTs compared to those in RCTs [10]. We also excluded in vitro and animal studies or studies in which research participants included non-first-time orthodontic patients. Studies in which treatment did not involve physical interventions, such as LLLT, vibration, chewing, and acupuncture; in which the outcome indicators did not include VAS scores; or from which data could not be extracted were excluded. Moreover, papers that were not written in Chinese or English were excluded.

### Study selection

Two trained reviewers (JX and SY) independently screened the titles and abstracts based on the inclusion criteria. The full texts of potentially eligible studies were assessed by the same reviewers. For studies in which the full text was inaccessible or those that had incomplete data, the original authors were contacted via email. If they could not be reached after three consecutive attempts or if the studies were confirmed to be inaccessible, the publications were excluded. In cases of discrepancies between the two reviewers, a third professional reviewer (LH) was consulted to achieve consensus.

### Data collection

We extracted the following data from the publications: author information, publication year, orthodontic correction method, type of vibration device, vibration frequency, vibration force, vibration time, laser frequency, laser power, laser irradiation time, chewing frequency, current intensity, current stimulation time, pain assessment timing, outcome indicators (VAS scores), and other relevant data.

### Quality assessment

Risk of bias was assessed by two authors (HJ and JZ), utilizing the Cochrane Handbook 5.1.0 version of the quality evaluation system [11], based on seven items to identify whether papers met the criteria for low, unclear, or high risk of bias. Any discrepancies were resolved through discussions with a third reviewer (YQ). The RoB2.0 risk bias summary chart [12] was generated using Review Manager 5.4 software (The Cochrane Collaborative, version 5.3, Cochrane IMS).

### Data analysis

Stata software (version 16.0; StataCorp LLC, College Station, TX, USA) was used to conduct the meta-analysis using the VAS score as the continuous outcome indicator. The mean difference (MD), standard deviation (SD), and 95% confidence interval (CI) were used as effect indicators. Heterogeneity between research results was determined using *I*^*2*^ (test level = 0.1), where *I*^*2*^>50% and *P*<0.1 indicated significant heterogeneity. In cases of significant heterogeneity, a random-effects model was employed, and sensitivity analysis was conducted using Stata v16.0. Subgroup analysis was performed using Review Manager 5.4 software. Statistical significance was set at *P*<0.05.

Stata v16.0 was also used to conduct a network meta-analysis. The “mvmeta” program was used to generate evidence network diagrams, evidence contribution diagrams, and funnel plots. To detect inconsistencies in the closed loop, the difference between the direct and indirect results was calculated. The inconsistency factor was determined using a Z-test, and the *P*-value of the Z-test was used to determine the presence of inconsistencies: *P*>0.05 indicated no inconsistency. Furthermore, the publication bias or small-sample effect of each intervention measure was determined separately, based on direct comparative data. The funnel plot, a tool utilized in Begg’s linear regression analysis, considered the magnitude and accuracy (in terms of the reciprocal of the standard error) of each study. A symmetric graph with a *P*>0.05 suggested absence of publication bias or small-sample effect in the meta-analysis.

The surface under the cumulative ranking curve (SUCRA) is a tool used to rank the effectiveness of different treatment methods and to determine the most optimal treatment plan. Its value ranges from 0 to 100. PrBest values also range between 0 and 100, with values closer to 100 indicating a greater likelihood of being the best intervention.

## Results

### Study selection

**Fig 1:**
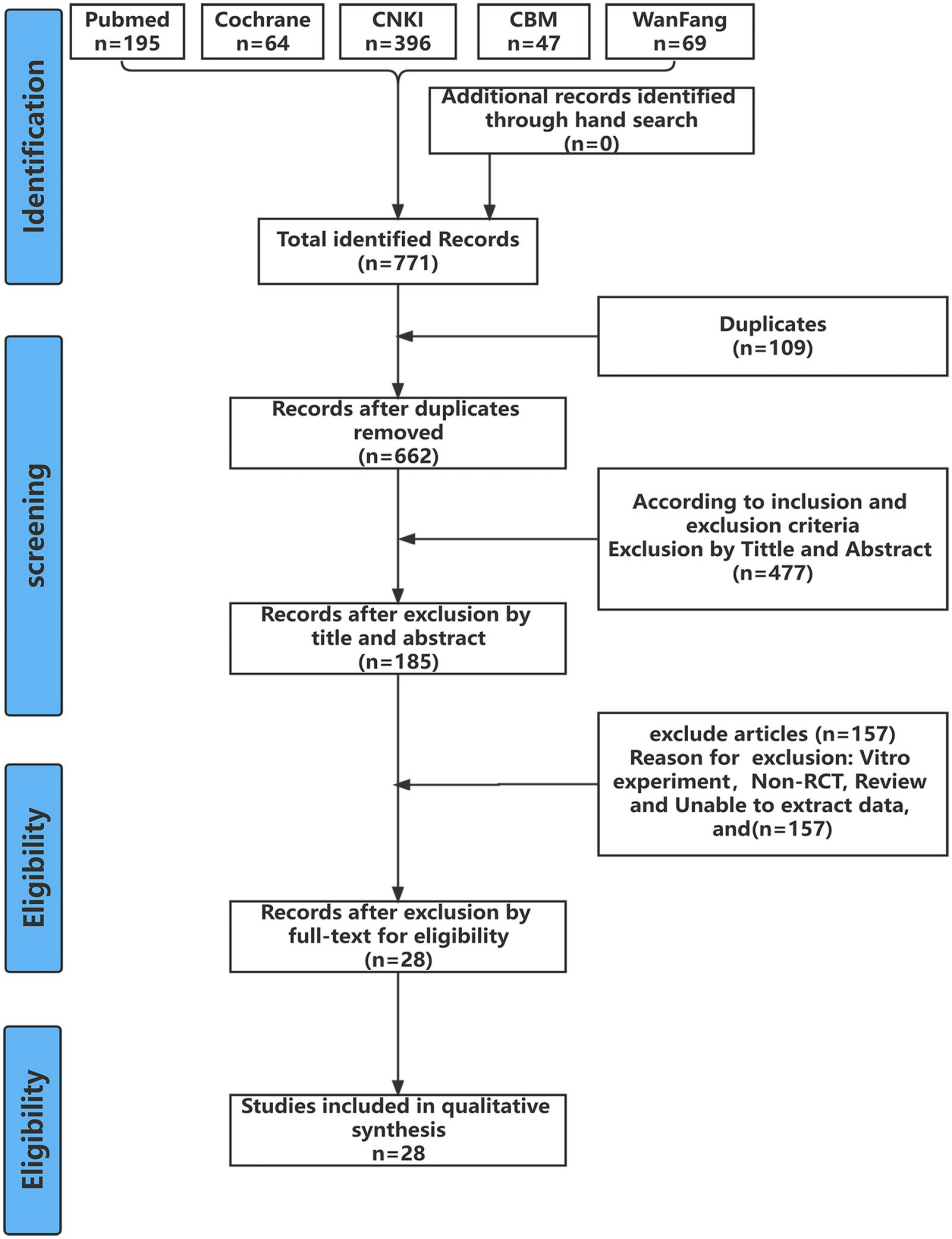
Flow chart of study selection.

The two researchers initially identified 771 relevant studies from various databases, including 277 articles on LLLT, 21 on vibration, 354 on chewing, and 120 on acupuncture. After comparing the search results of the major databases, 109 duplicate articles were excluded. After carefully reading the titles and abstracts, 477 irrelevant articles were excluded. After assessing the full text, 157 articles were excluded as they reported on in vitro experiments, non-RCTs, reviews, systematic evaluations, and meta-analyses or did not allow extraction of effective data. Finally, 28 articles [13–40] were included. The literature screening process is illustrated in Fig 1.

### Study characteristics

The 28 included articles [13–40] measured the VAS score as outcome indicator at 24 h after initiating orthodontic treatment, while 22 articles [14–20, 22–24, 26–29, 32, 34–40] measured this outcome indicator at 48 h. Table 2 shows the characteristics of the included studies.

**Table 2:** Characteristics of included studies.

Study [13–40]	Country	Sample size	Intervention measures	Outcome measures (VAS)
Mirhashemi et al. 2021 [13]	Iran	E = 26; C = 26	E: LLLT; C: Placebo	24 h: E: 72.2 ± 19.5/C: 79.2 ± 18.5
Martins 2019 [14]	Brazil	E = 64; C = 64	E: LLLT; C: Placebo	24 h: E: 16 ± 18/C: 18 ± 20 48 h: E: 8 ± 14/C: 11 ± 14
Qamruddin et al. 2017 [15]	Pakistan	E = 20; C = 20	E: LLLT; C: Placebo	24 h: E: 14 ± 8.2/C: 22 ± 4.1 48 h: E: 10 ± 6.4/C: 14 ± 10.4
Kim et al. 2013 [16]	South Korea	E = 28; C = 30	E: LLLT; C: Placebo	24 h: E: 26.64 ± 6.28/C: 50.47 ± 5.62 48 h: E: 26.59 ± 6.28/C: 47.11 ± 5.63
Prasad et al. 2019 [17]	India	E = 10; C = 10	E: LLLT; C: Placebo	24 h: E: 12 ± 17/C: 25 ± 9.7 48 h: E: 6 ± 8.4/C: 12 ± 10.3
Stein et al. 2015 [18]	Germany	E = 20; C = 20	E: LLLT; C: Placebo	24 h: E: 20 ± 18/C: 46 ± 34 48 h: E: 20 ± 20/C: 32 ± 26
Almallah et al 2020 [19]	Syria	E = 18; C = 18	E: LLLT; C: Placebo	24 h: E: 22.61 ± 28.16/C: 33.28 ± 31.7 48 h: E: 19.28 ± 24.59/C: 31.22 ± 29.6
AlSayed Hasan et al. 2020 [20]	Syria	E = 13; C = 13	E: LLLT; C: Placebo	24 h: E: 14.92 ± 12.48/C: 22 ± 24.28 48 h: E: 10.23 ± 9.98/C: 15.23 ± 21.01
Eslamian et al. 2014 [21]	Iran	E = 74; C = 74	E: LLLT; C: Placebo	24 h: E: 10.4 ± 17.2/C: 14.2 ± 2.25
Qamruddin et al. 2018 [22]	Pakistan	E = 42; C = 42	E: LLLT; C: Placebo	24 h: E: 20 ± 16/C: 35 ± 26 48 h: E: 33 ± 29/C: 52 ± 31
Almallah et al. 2016 [23]	Syria	E = 18; C = 18	E: LLLT; C: Placebo	24 h: E: 27.22 ± 20.99/C: 41.78 ± 24 48 h: E: 20.11 ± 21.85/C: 30.28 ± 23.89
Ren et al. 2020 [24]	China	E = 27; C = 27	E: LLLT; C: Placebo	24 h: E: 8.1 ± 13.1/C: 15 ± 21.9 48 h: E: 4.2 ± 8.9/C: 6.9 ± 12.6
Domínguez and Velásquez 2013 [25]	Columbia	E = 30; C = 30	E: LLLT; C: Placebo	24 h: E: 3.26 ± 0.368/C: 6.96 ± 0.315
Qamruddin et al. 2016 [26]	Pakistan	E = 88; C = 88	E: LLLT; C: Placebo	24 h: E: 18.6 ± 25/C: 50.4 ± 32.4 48 h: E: 15.4 ± 23.7/C: 45.4 ± 32.7
Guram et al. 2018 [27]	India	E = 20; C = 20	E: LLLT; C: Placebo	24 h: E: 18.8 ± 22.1/C: 51.6 ± 48.6 48 h: E: 12.1 ± 31.2/C: 22.3 ± 28.9
Ghaffar et al. 2022 [28]	Egypt	E = 15; C = 15	E: LLLT; C: Placebo	24 h: E: 30 ± 20/C: 20 ± 9 48 h: E: 39 ± 20/C: 30 ± 16
Katchooi et al. 2018 [29]	America	E = 13; C = 13	E: Vibration; C: Control	24 h: E: 34 ± 24/C: 47 ± 31 48 h: E: 33 ± 19/C: 28 ± 3.9
Alansari et al. 2018 [30]	America	E = 14; C = 13	E: Vibration; C: Control	24 h: E: 33.9 ± 13.5/C: 41.9 ± 7.1
Feng et al. 2016 [31]	China	E = 20; C = 20	E: Vibration; C: Control	24 h: E: 41.54 ± 14.21/C: 57.12 ± 21.89
Deng et al. 2019 [32]	China	E = 30; C = 30	E: Vibration; C: Control	24 h: E: 44.7 ± 7.2/C: 55.6 ± 8.9 48 h: E: 34.2 ± 8.3/C: 47.5 ± 8.9
Miles et al. 2012 [33]	Australia	E = 30; C = 28	E: Vibration; C: Control	24 h: E: 41.5 ± 27.2/C: 47.6 ± 24.5
Celebi 2022 [34]	Turkey	E = 19; C = 19	E: Chewing; C: Vibration	24 h: E: 52.1 ± 24.2/C: 40.3 ± 29.5 48 h: E: 42.8 ± 20.8/C: 32.5 ± 24.9
Yang et al. 2013 [35]	China	E = 50; C = 64	E: Chewing; C: Control	24 h: E: 14.56 ± 12/C: 30.34 ± 25.91 48 h: E: 11.42 ± 12.01/C: 24.83 ± 22.32
Celebi et al. 2021 [36]	Turkey	E = 21; C = 21	E: LLLT; C: Chewing	24 h: E: 48 ± 21.2/C: 51.6 ± 20.8 48 h: E: 42 ± 23.7/C: 42.7 ± 19.3
Farzanegan et al. 2012 [37]	Iran	E = 10; C = 10	E: Chewing; C: Control	24 h: E: 34.7 ± 38.3/C: 74.7 ± 27.3 48 h: E: 38 ± 33.9/C: 66.4 ± 31.1
Roth and Thrash 1986 [38]	America	E = 9; C = 9	E: TENS; C: Control	24 h: E: 4.77 ± 6.96/C: 14.55 ± 6.89 48 h: E: 5.55 ± 9.3/C: 16.44 ± 8.24
Jia et al. 2016 [39]	China	E = 23; C = 20	E: TEAS; C: Control	24 h: E: 29.1 ± 6.7/C: 40.5 ± 10.5 48 h: E: 34.8 ± 9.9/C: 58 ± 11.5
Zhao 2018 [40]	China	E = 45; C = 45	E: Classical acupuncture; C: Control	24 h: E: 15.85 ± 14/C: 41.1 ± 20.5 48 h: E: 10.9 ± 11.8/C: 32.1 ± 17.21

E, experimental group; C, control group; VAS, visual analog scale; LLLT, low-level laser therapy; TENS, transcutaneous electrical nerve stimulation; TEAS, transcutaneous electrical acupoint stimulation

### Risk of bias within studies

**Fig 2:**
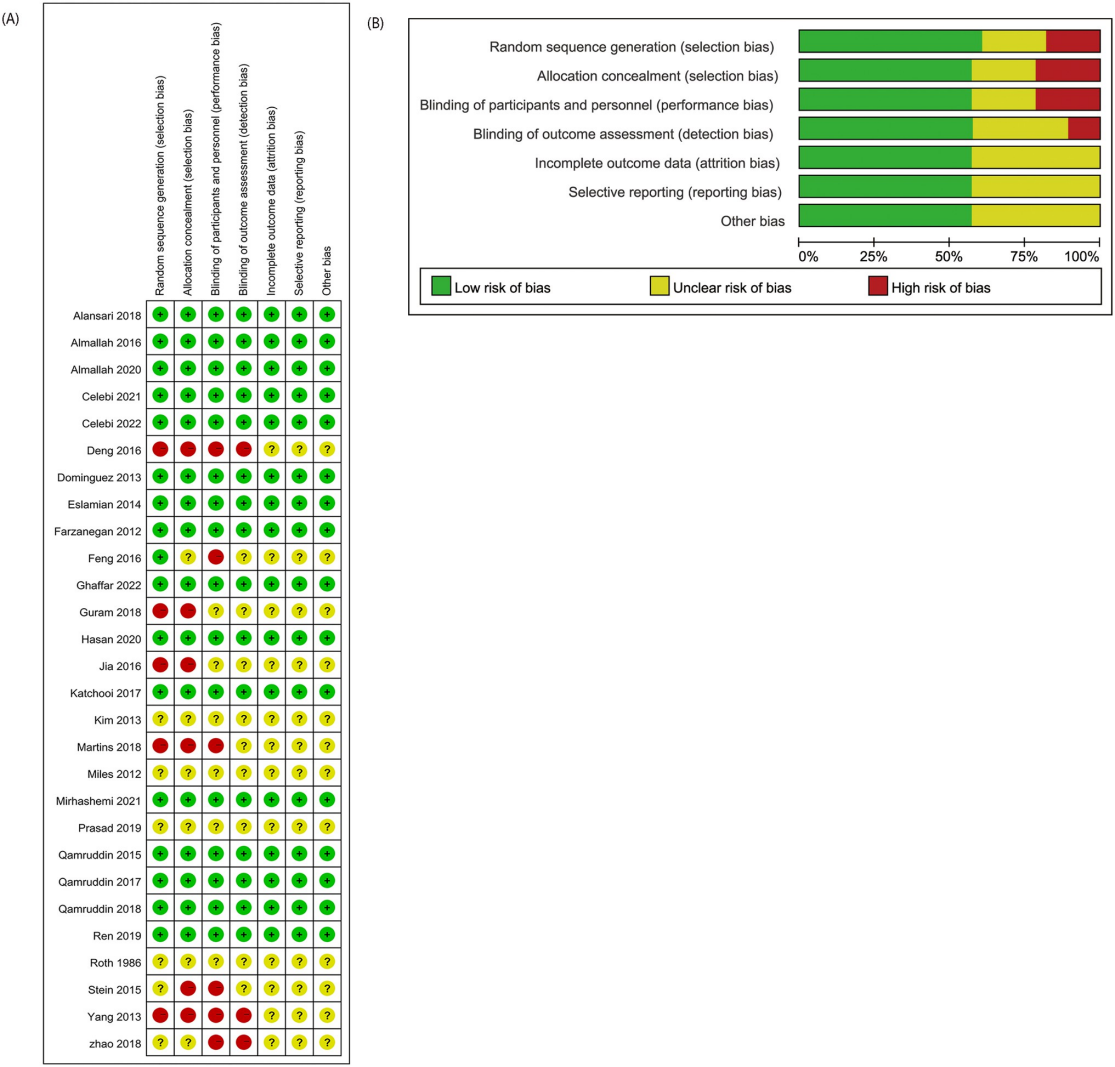
A. Risk of bias summary. B. Risk of bias graph.

The specific experimental details of the included studies are shown in Table 3. Among the included studies, 17 had low risk of bias [13–15, 19–26, 28–31, 36, 37], four had unclear risk of bias [16, 17, 33, 38], and seven had high risk of bias [18, 27, 31–33, 39, 40], as shown in Fig 2A and 2B.

**Table 3:** Implementation details of the studies.

Study [13–40]	Treatment	Age (years)	Intervention details
Mirhashemi et al. 2021 [13]	Place elastic separators on the mesial and distal adjacent surface of the maxillary first molar	13–37	LLLT-diode laser 400 W, 15.6 J·cm^-2^, 11 s × 2 = 22 s
Martins et al. 2019 [14]	Place elastic separators on the mesial and distal adjacent surface of the mandibular first molar	average 19.8	LLLT-diode laser 100 mW, 95 J·cm^-2^, 3 J, 30 s × 8 = 240 s
Qamruddin et al. 2017 [15]	MBT self-ligating brackets + 0.019" × 0.025" stainless steel wire + 150 g force retracts canines	12–25	Gallium–aluminum–arsenic diode laser 100 mW, 940 nm, 7.5 J·cm^-2^, 3 s × 10 = 30 s
Kim et al. 2013 [16]	Place elastic separators on the mesial and distal adjacent surface of the maxillary first molar	average 22.7	AlGalnP diode laser 6 mW, 635 nm, 30 s × 2 = 60 s
Prasad et al. 2019 [17]	Traditional ligating brackets + 0.019" × 0.025" stainless steel wire 200 g force retracts canines	18–24	Diode laser 600 J, 980 nm, 2.5 W·cm^-2^, 30 s × 2 = 60 s
Stein et al. 2015 [18]	Place elastic separators on the mesial adjacent surface of the mandibular first molar	6–9	Diode laser 100 mW, 660 nm, 20 s × 10 = 200 s
Almallah et al. 2020 [19]	Place elastic separators on the mesial and distal adjacent surface of the maxillary first molar	12–25	GaAlAs diode laser 100 mW, 830 nm, 4 J·cm^-2^, 28 s × 8 = 224 s
AlSayed Hasan et al. 2020 [20]	MBT traditional ligating brackets + 0.014" Ni-Ti round wire	16–24	LLLT 150 mW, 830 nm, 2 J, 60 s × 6 = 360 s
Eslamian et al. 2014 [21]	Place elastic separators on the mesial and distal adjacent surface of the maxillary first molar	11–32	GaAlAs diode laser 100 mW, 810 nm, 2 J·cm^-2^, 20 s × 10 = 200 s
Qamruddin et al. 2018 [22]	MBT brackets + 0.014" Ni-Ti round wire	12–25	Al-Ga-As diode laser, 100 mW, 940 nm, 7.5 J·cm^-2^, 3 s × 60 = 210 s
Almallah et al. 2016 [23]	Place elastic separators on the mesial adjacent surface of the mandibular first molar	12–26	GaAlAs diode laser 100 mW, 830 nm, 4 J·cm^-2^, 28 s × 8 = 224 s
Ren et al. 2020 [24]	Straight wire arch brackets + 0.014" Ni-Ti round wire	31–60	GaAlAs diode laser 800 mW, 940 nm, 8.6 J·cm^-2^, 30 s(Cover the entire arch)
Domínguez and Velásquez 2013 [25]	GAC self-ligating + 0.019" × 0.025" stainless steel square wire	20–30	GaAlAs laser 100 mW, 830 nm, 80 J·cm^-2^, 22 s (Cover the entire arch)
Qamruddin et al. 2016 [26]	Place elastic separators on the mesial adjacent surface of the mandibular first molar	13–30	GaAlAs laser 200 mW, 940 nm, 4 J·cm^-2^, 20 s × 3 = 60 s
Guram et al. 2018 [27]	MBT self-ligating brackets + 0.019" × 0.025" Stainless steel wire + 150 g force retracts canines	17–24	GaAlAs laser 200 mW, 810 nm, 5 J·cm^-2^, 5 s × 6 = 30 s
Ghaffar et al. 2022 [28]	Roth brackets + 0.014" Ni-Ti round wire	18–25	LLLT-diode laser 25.7 J·cm^-2^ (No other parameters presented)
Katchooi et al. 2018 [29]	Invisible appliance	≥ 18	Acceledent, 30 Hz, 25 g, 20 min
Alansari et al. 2018 [30]	Invisible appliance	18–45	Vpro5, 120 Hz, 0.03 g, 5 min
Feng et al. 2016 [31]	MBT straight wire appliance + 0.016" Ni-Ti round wire	Children	Acceledent, 30 Hz, 25 g, 20 min
Deng et al. 2019 [32]	Fixed appliance	6–14	Acceledent, 30 Hz, 25 g, 20 min
Miles et al. 2012 [33]	MBT straight wire appliance + 0.014" Ni-Ti round wire	11–15	Tooth Masseuse, 111 Hz, 6.1 g, 20 min
Celebi 2022 [34]	Roth brackets + 0.014" Ni-Ti round wire	12–24	Vibration: 111 Hz, 0.06 N, 60 min; Chewing gum 20 min
Yang et al. 2013 [35]	IMD brackets + 0.014" Ni-Ti round wire	> 10	Chewing gum (No other parameters presented)
Celebi et al. 2021 [36]	Roth brackets + 0.014" Ni-Ti round wire	12–24	GaAlAs diode laser 50 mW, 820 nm, 0.8 J·cm^-2^, 30 s × 72 = 1152 s; Chewing gum 20 min × 3 = 60 min
Farzanegan et al. 2012 [37]	Fixed appliance + 0.016" Ni-Ti round wire	13–18	Chewing gum 5 min × 3 = 15 min
Roth and Thrash 1986 [38]	Place elastic separators on the mesial adjacent surface of the maxillary first molar	22–41	TENS: 0.5 Hz, 500 mA, 20 min
Jia et al. 2016 [39]	Edgewise appliance + 0.014" Ni-Ti round wire	13–23	TEAS: 20–100 Hz, 30 min × 2 = 60 min
Zhao 2018 [40]	MBT straight wire appliance + 0.013" Ni-Ti round wire	18–33	Classical acupuncture 10–15 min

LLLT, low-level laser therapy; TENS, transcutaneous electrical nerve stimulation; TEAS, transcutaneous electrical acupoint stimulation

### Effects of pain control intervention after orthodontic treatment

#### LLLT

In terms of the use of LLLT, 17 articles [13–28, 36] included VAS scores at 24 h after initiating orthodontic treatment, while 14 articles [14–20, 22–24, 26–28, 36] included the VAS score at 48 h after initiating orthodontic treatment. The total sample size was 810. Heterogeneity was significant at both 24 and 48 h after treatment; therefore, a random-effects model was chosen. The difference between 24 h (MD = -1.07, 95% CI [-1.56, -0.58], *P*<0.001) (Table 4, S1 Fig) and 48 h (MD = -0.58, 95% CI [-0.94, -0.22], *P* = 0.002) (Table 5, S2 Fig) was statistically significant. Thus, LLLT intervention resulted in a significant decrease in VAS pain scores of patients at 24 and 48 h after initiating orthodontic treatment.

**Table 4:** Meta-analysis results of different interventions after 24 h.

Interventions	Reference no	Heterogeneity		Effect model	Meta analysis results		
		*I* ^*2*^	*P*		*Z*	95% CI (%)	*P*
LLLT	[13–28, 36]	92.3%	*P*<0.001	Random-effects model	-1.07	(-1.56, -0.58)	*P*<0.001
Vibration	[29–34]	45.5%	0.102	Random-effects model	-0.69	(-1.04, -0.33)	*P*<0.001
Chewing	[34–37]	61.1%	0.052	Fixed-effects model	-0.51	(-0.79, -0.24)	*P*<0.001
Acupuncture	[38–40]	10.9%	0.326	Fixed-effects model	-1.48	(-1.85, -1.12)	*P*<0.001

LLLT, low-level laser therapy; CI, confidence interval

**Table 5:** Meta-analysis results of different interventions after 48 h.

Interventions	Studies	Heterogeneity		Effect model	Meta analysis results		
		*I* ^*2*^	*P*		*Z*	95% CI (%)	*P*
LLLT	[14–20, 22–24, 26–28, 36]	82.3%	*P*<0.001	Random-effects model	-0.58	(-0.94, -0.22)	*P*<0.001
Vibration	[30, 33, 35]	87.4%	*P*<0.001	Fixed-effects model	-0.73	(-1.11, -0.36)	*P*<0.001
Chewing	[34–37]	45.7%	0.137	Fixed-effects model	-0.48	(-0.76, -0.21)	*P*<0.001
Acupuncture	[38–40]	32.3%	0.228	Fixed-effects model	-1.67	(-2.05, -1.30)	*P*<0.001

LLLT, low-level laser therapy; CI, confidence interval

#### Vibration

In terms of the use of vibration for orthodontic pain, six studies [29–34] calculated the VAS scores at 24 h and three studies [29, 32, 34] measured VAS scores at 48 h after initiating orthodontic treatment. The total sample size was 249. Heterogeneity was significant at 24 h after treatment; therefore, a random-effects model was used. However, due to the limited number of studies, a fixed-effect model was used to evaluate the effect of vibration at 48 h [41]. The difference between the postoperative pain levels at 24 h (MD = -0.69, 95% CI [-1.04, -0.33], *P*<0.001) (Table 4, S3 Fig) and at 48 h (MD = -0.73, 95% CI [-1.11, -0.36], *P*<0.001) (Table 5, S4 Fig) was statistically significant, indicating that vibration therapy can alleviate the severe pain associated with orthodontic treatment.

#### Chewing

Four studies evaluated the effect of chewing on the VAS pain scores at 24 h and 48 h after initiating orthodontic treatment [34–37]. The final sample size was 214. As only four studies with limited available research data were included, the Mantel–Haenszel method with a fixed effects model was used. The difference between 24 h (MD = -0.51, 95% CI [-0.79, -0.24], *P*<0.001) (Table 4, S5 Fig) and 48 h (MD = -0.48, 95% CI [-0.76, -0.21], *P*<0.001) (Table 5, S6 Fig) was statistically significant. These findings suggest that chewing therapy can effectively alleviate pain following orthodontic treatment.

#### Acupuncture

Three studies with a sample size of 151 assessed the VAS pain scores at 24 and 48 h after initiating orthodontic treatment [38–40]. The heterogeneity test results showed low heterogeneity; thus, the Mantel–Haenszel test with a fixed effects model was used. We found a statistically significant difference between 24 h ([MD = -1.48, 95% CI (-1.85, -1.12), *P*<0.001) (Table 4, S7 Fig) and 48 h (MD = -1.67, 95% CI (-2.05, -1.30), *P*<0.001) (Table 5, S8 Fig) in terms of relief of pain sensation. It can be concluded that acupuncture can significantly relieve pain sensation after orthodontic treatment.

### Network meta-analysis

**Fig 3:**
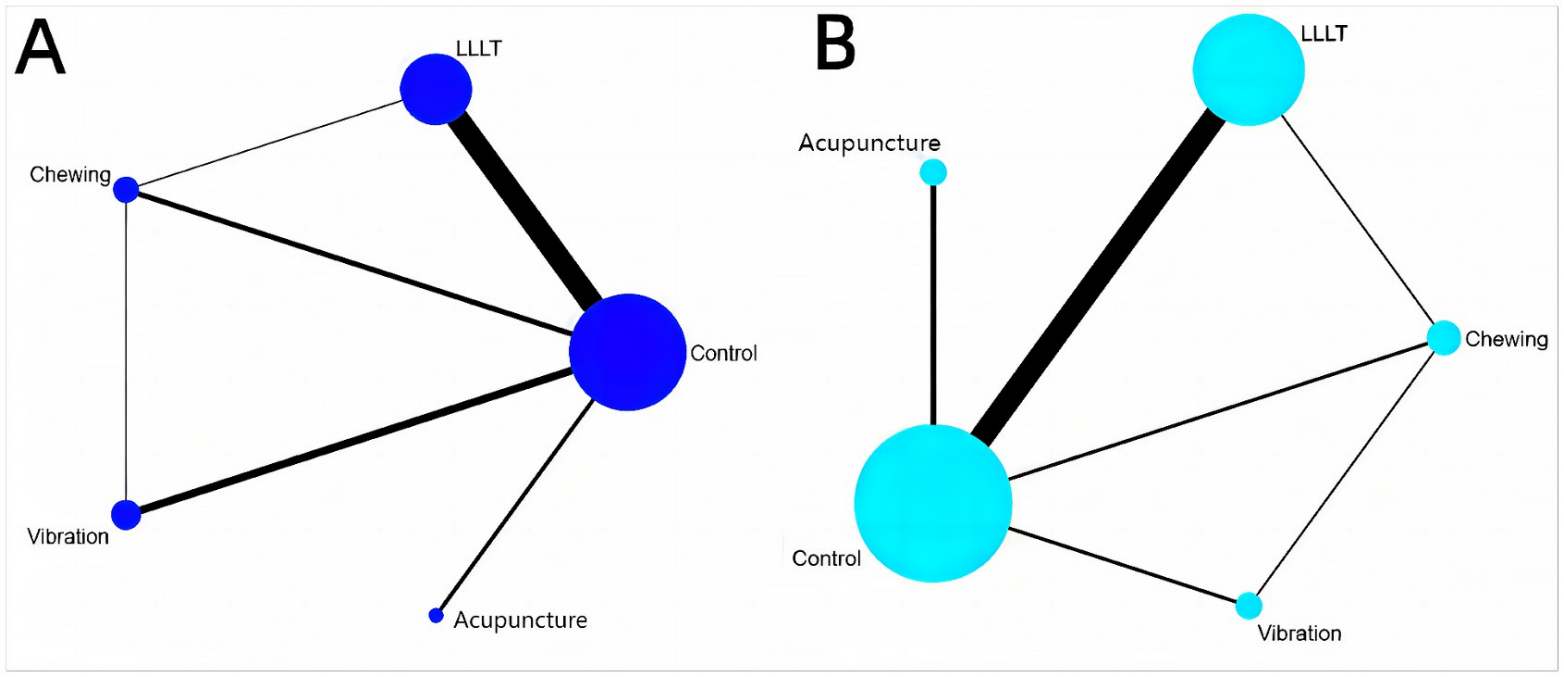
Network graphs for various outcomes. (A) Outcomes at 24 h after treatment; (B) Outcomes at 48 h after treatment.

Fig 3A and 3B illustrate the network structure at two different time points. Ten pairs of comparisons were made between the two time points. Six pairs were directly compared, whereas four pairs of indirect comparisons were generated through a network meta-analysis (acupuncture vs. LLLT, acupuncture vs. chewing, acupuncture vs. vibration, LLLT vs. vibration). The control group had the largest sample size, followed by the LLLT group.

**Fig 4:**
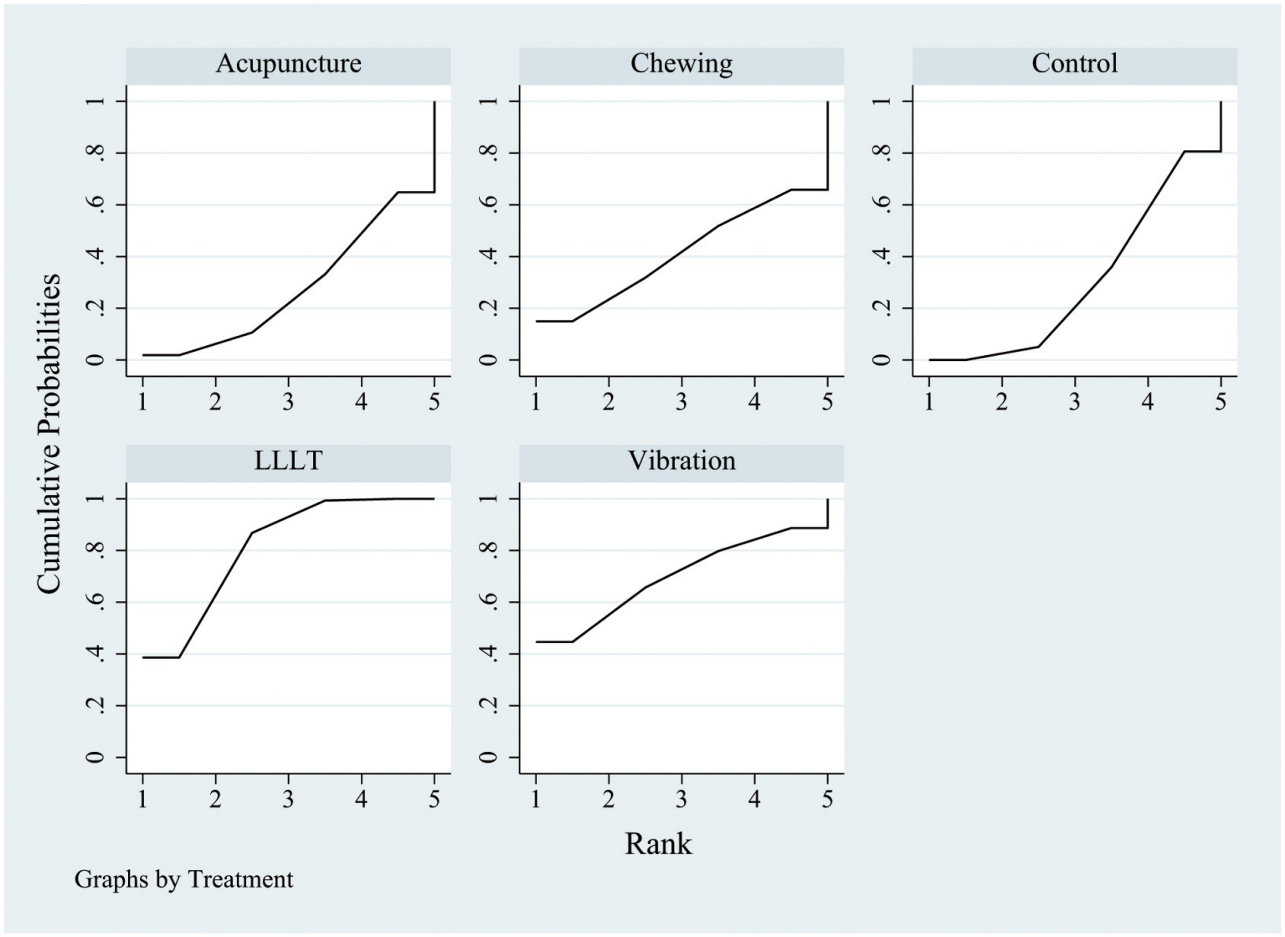
SUCRA probability ranking after 24 h of treatment. SUCRA, surface under the cumulative ranking curve.

After 24 h of physical intervention, the sorting chart (Fig 4) and sorting results (Table 6) showed that the SUCRA value was the highest in the LLLT group (80.8), followed by the vibration therapy group (71.1), and the lowest in the control group (30.1). The PrBest value was the highest in the LLLT group (38.2), followed by the vibration therapy group (45.7), and 0 in the control group. The MeanRank value showed that LLLT and vibration therapy ranked as the top two treatments. Based on these results, it can be concluded that LLLT and vibration therapy are effective intervention measures.

**Table 6:** Sorting results after 24 h of treatment.

Treatment (24 h)	SUCRA	PrBest	MeanRank
Control	30.1	0.0	3.8
LLLT	80.8	38.2	1.8
Chewing	41.0	14.6	3.4
Vibration	71.1	45.7	2.2
Acupuncture	27.0	1.5	3.9

SUCRA, surface under the cumulative ranking curve; LLLT, low-level laser therapy

**Fig 5:**
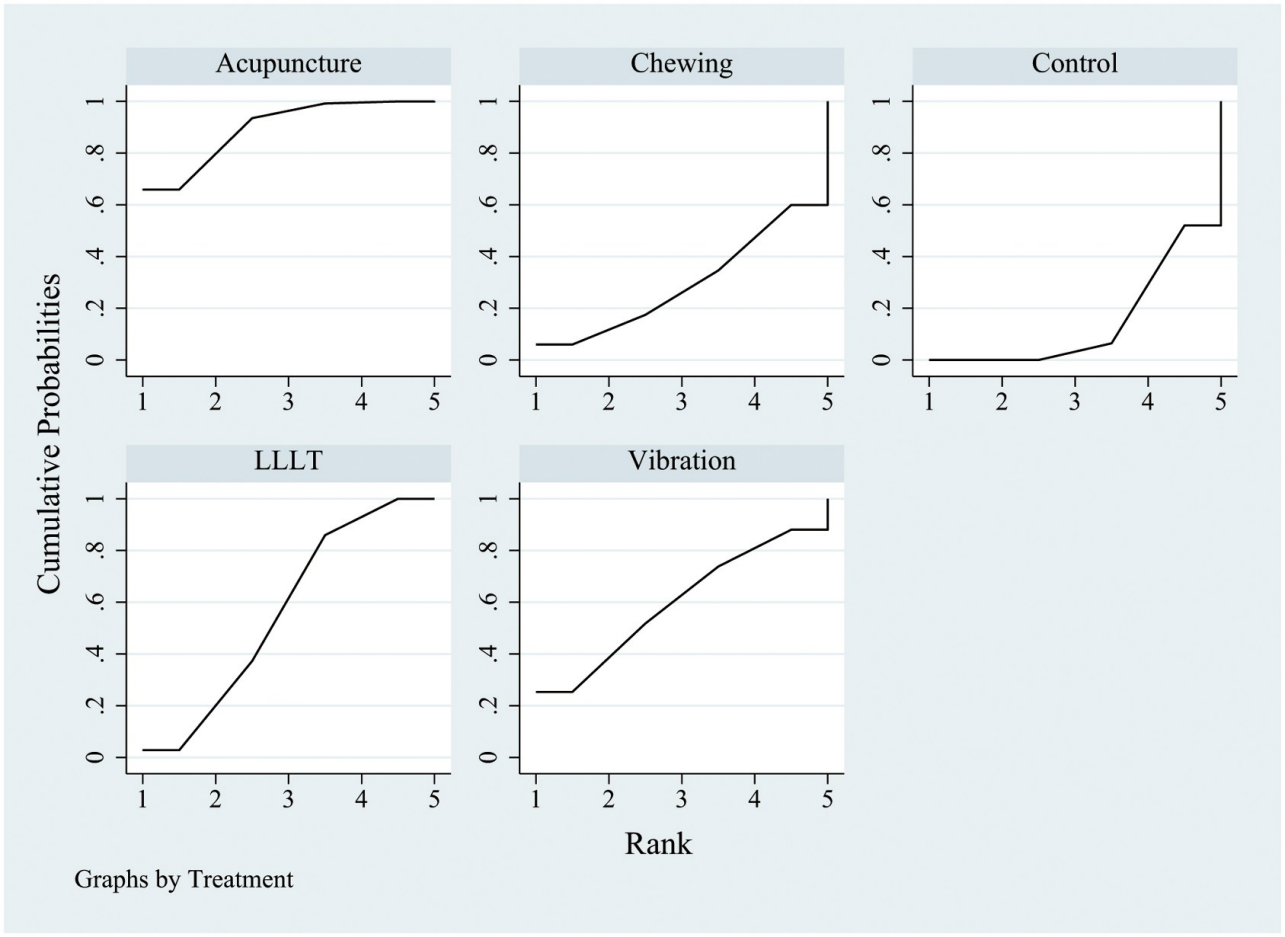
SUCRA probability ranking after 48 h of treatment. SUCRA, surface under the cumulative ranking curve.

After 48 h of implementation of various physical interventions, the results indicated that acupuncture had the highest SUCRA value (89.6), followed by vibration therapy (59.7); the control group had the lowest value (14.6) (Fig 5, Table 7). Additionally, the PrBest value was the highest for acupuncture (65.9), followed by vibration therapy (25.3); the control group had no value. Furthermore, the MeanRank values showed that acupuncture and vibration therapy were the top two interventions. Acupuncture was found to be the most effective in relieving pain after 48 h of treatment.

**Table 7:** Sorting results after 48 h of treatment.

Treatment (48 h)	SUCRA	PrBest	MeanRank
Control	14.6	0.0	4.4
LLLT	56.5	2.8	2.7
Chewing	29.5	6.0	3.8
Vibration	59.7	25.3	2.6
Acupuncture	89.6	65.9	1.4

SUCRA, surface under the cumulative ranking curve; LLLT, low-level laser therapy

### Sensitivity analysis

**Fig 6:**
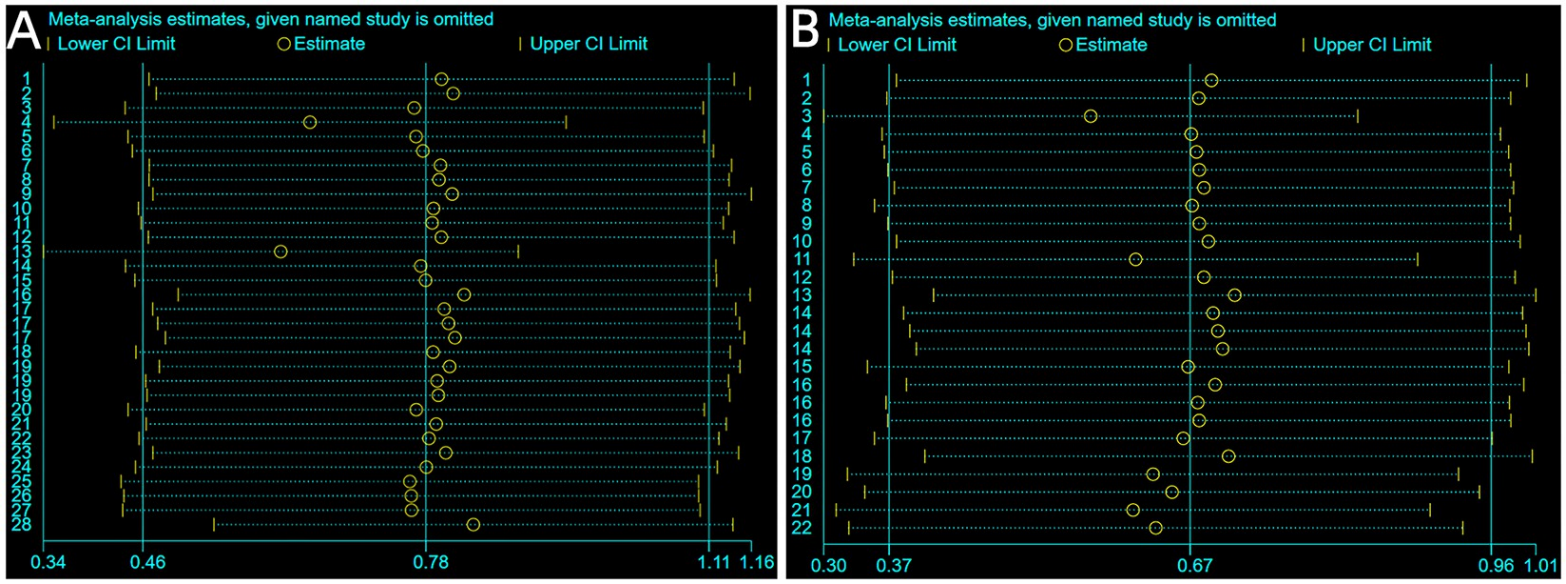
Sensitivity analysis. (A) Outcomes at 24 h after treatment; (B) Outcomes at 48 h after treatment.

For sensitivity analysis, we utilized a leave-one-out exclusion method to analyze the outcome indicators of VAS scores at 24 and 48 h after treatment. Each study was excluded individually before merging the effects. Sensitivity analysis did not affect the results. This indicated that the statistical analysis results of the merged effects in this meta-analysis were stable and reliable, as shown in Fig 6A and 6B.

### Publication bias

**Fig 7:**
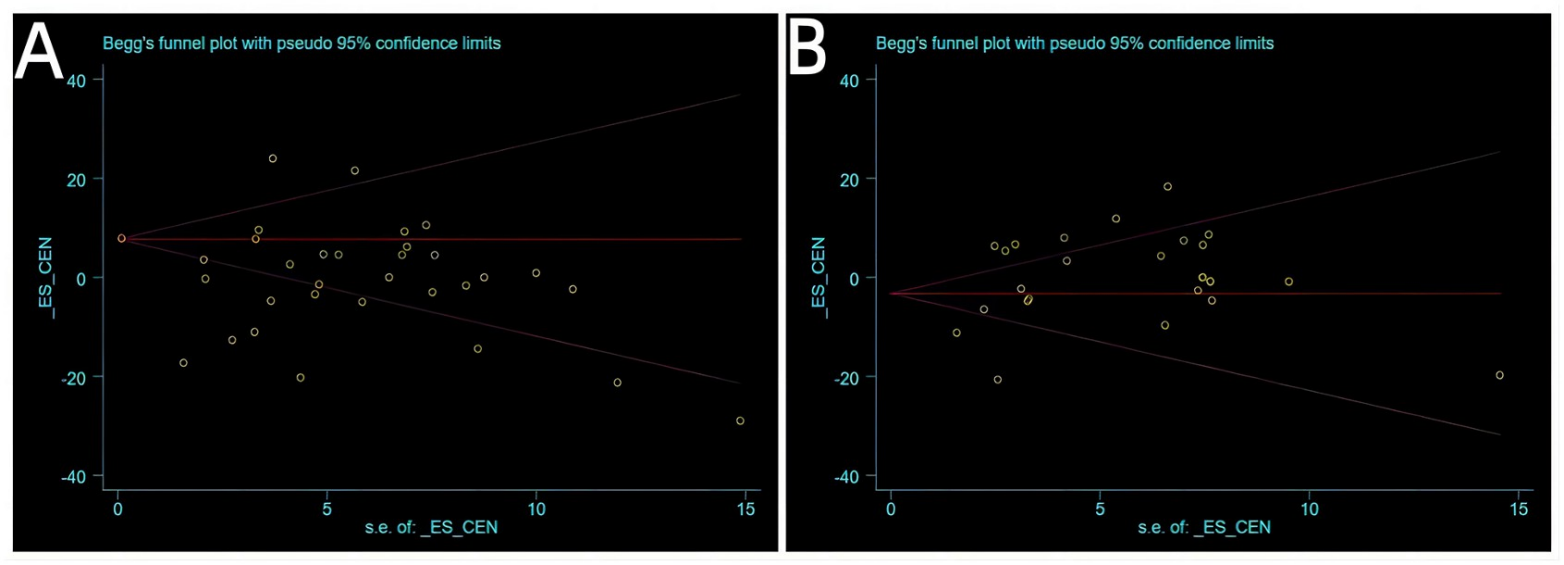
Funnel plots for publication bias. (A) Outcomes at 24 h after treatment; (B) Outcomes at 48 h after treatment.

Begg’s linear regression analysis was conducted for outcomes at 24 and 48 h of treatment. The results yielded *P* values of 0.466 and 0.537, respectively, which both exceeded the significance level of 0.05. This indicated the absence of publication bias during the study (Fig 7A and 7B).

## Discussion

According to previous research, approximately 30% of patients who do not achieve their orthodontic goals and terminate treatment early do so because they cannot tolerate the pain [42]. The results of this meta-analysis demonstrated that vibration therapy, LLLT, chewing therapy, and acupuncture could effectively reduce pain scores measured using the VAS at 24 and 48 h after initiating orthodontic treatment. Specifically, acupuncture was found to be the most effective intervention to alleviate pain within 48 h of commencing orthodontic treatment.

Orthodontic force mechanically compresses the periodontal tissue, which hinders local blood circulation and releases inflammatory factors, neuropeptides, and other pain response-mediating factors, ultimately causing non-infectious inflammation [43]. Pain signal transduction involves multiple ion channels, including acid-sensing ion channel-3 (ASIC3) and transient receptor potential vanilloid 1 (TRPV1), which play vital roles in mediating orthodontic pain [44]. Orthodontic force compresses the periodontal tissue capillaries on the pressure side, resulting in local ischemia and hypoxia. This change increases anaerobic respiration in the periodontal tissue and creates an acidic microenvironment, which stimulates the opening of the ASIC3 and TRPV1 channels. These channels are highly sensitive to acidic environments (H^+^) and allow a large amount of Ca^2+^ to enter neurons, triggering nerve impulses that signal pain [44, 45]. Pain signals are carried to the cerebral cortex via the trigeminal ganglion and neuron bodies in the trigeminal caudate nucleus, which then generates pain perception [46].

Postoperative pain relief methods are typically categorized as psychotherapy, physical therapy, and drug therapy. Examples of these methods include music, self-suggestion, attention transfer, low-dose lasers, acupoint stimulation, nonsteroidal anti-inflammatory drugs (NSAIDs), traditional Chinese medicine, and other treatments [47]. Several studies have reported that NSAIDs can impede the synthesis of prostaglandins, which can lead to a decrease in the inflammation caused by periodontal ligament cells and hinder osteoclast production. Although NSAIDs can alleviate postoperative pain, they can also cause side effects that can reduce tooth movement speed by approximately 50% [48]. Moreover, indiscriminate use of NSAIDs can cause gastrointestinal bleeding [49]. The drawbacks of traditional pain-relief methods, such as uncertain efficacy, strong invasiveness, significant side effects, and low patient compliance, have made it necessary to find more efficient, safe, and comfortable methods for relieving the pain caused by orthodontic treatment.

The VAS is considered to be the most reliable method for evaluating pain perception [50]. Therefore, the VAS score was used as an outcome indicator in this meta-analysis. Pain typically occurs approximately 3 h after applying force following orthodontic surgery [51], although the timing of the most severe pain remains debated. However, severe pain is mainly concentrated between 24 and 48 h after commencing treatment [52]. Thus, this study focused on literature describing the degree of pain experienced during this period after orthodontic treatment.

The literature on vibration therapy included in this study showed significant heterogeneity, which may be attributed to the diverse types of vibrators used as well as variations in vibration force, frequency, and duration. These factors have a notable impact on VAS scores. The analgesic effects of mechanical vibration may result from the activation of cyclic guanosine 3’,5’-monophosphate-dependent Na/Ca exchange in the forward mode [11]. Vibration stimulation can decrease the levels of TRPV1 and calcitonin gene-related peptide (CGRP) in the periodontal tissue after orthodontic surgery [53]. CGRP is a neuropeptide found in the periodontal tissue and is responsible for promoting pain after surgery [53]. Studies have focused on regulating CGRP through targets such as TRPV1 and nociceptin/orphanin FQ [54]. Evidence-based medical research has also shown that high-frequency vibrations can accelerate orthodontic tooth movement by promoting the differentiation of osteoblasts, fibroblasts, and osteoclasts and increasing bone remodeling; thereby accelerating the process [55, 56]. In addition, high-frequency vibrations can reduce the risk of root resorption after orthodontic treatment [57]. The research included in this study involved the use of Accelent and Vpro5 vibrators. In a comparative study conducted by Judex et al. [58], both Vpro5 and Accellent vibrators increased the proliferation and gene expression of osteoblasts, fibroblasts, and osteoclasts. However, this study showed that response to treatment was stronger with Vpro5 than with Accellent, which may be attributed to the former’s high vibration frequency. Vpro5 has a higher vibration frequency and smaller vibration amplitude than those of Accellent, making it more comfortable to use. However, the exact mechanism by which vibration reduces orthodontic pain is not fully understood. Additionally, the ideal parameters for achieving a balance between accelerated tooth movement and pain relief are still debated.

This network meta-analysis found that most studies on LLLT used diode lasers; however, the studies demonstrated significant heterogeneity. The main sources of heterogeneity may be the laser power, wavelength, irradiation range, frequency, and time. LLLT is widely used in oral clinical diagnosis and treatment because of its unique advantages in oral mucosal hemostasis, analgesia, and the promotion of periodontal tissue healing [59]. LLLT has several advantages as an orthodontic pain treatment. It has no serious side effects, causes minimal trauma, and can be used repeatedly. Several studies have demonstrated the effectiveness of LLLT in reducing orthodontic pain and improving the tooth movement rate [60]. According to Domínguez et al. [61], LLLT reduces the average duration of orthodontic treatment by approximately 24%. Furthermore, the acceleration of tooth movement resulting from multiple laser irradiations can be maintained throughout the treatment process. LLLT also has a positive effect on the local inflammation level of the periodontal tissue within 24 h of irradiation. LLLT irradiation can reduce the expression levels of tumor necrosis factor-α, cyclooxygenase 2, and prostaglandin E2 [62]. LLLT has been found to reduce the speed of axonal flow and decrease the membrane potential of mitochondria by vascularizing the peripheral nerves in the periodontal tissue. This reduction in vascularization leads to a decrease in the availability of adenosine triphosphate, which in turn impedes signaling in class A/C nerve fibers [63]. Dhobley et al. [64] discovered that LLLT can increase the production of β-endorphins, which are natural mediators that help reduce pain. Moreover, LLLT inhibits the release of arachidonic acid, which causes damaged cells to produce metabolites that interact with pain receptors [65]. This meta-analysis included studies that focused on the wavelength of LLLT, which was primarily concentrated above 600 nm. The optimal wavelength range for promoting orthodontic tooth movement through LLLT is between 780 nm and 830 nm, as suggested in previous research [61]. Additionally, LLLT at 830 nm has been found to have superior analgesic effects for orthodontic treatment as compared to other laser wavelengths [66].

TEAS is a novel therapeutic method that combines TENS, which is often used in sports medicine, with acupoint stimulation in Chinese acupuncture. By applying microcurrents through the skin and into acupoints, TEAS stimulates the meridian system and enhances the internal function [67]. Low-frequency (2 Hz) TEAS has been found to enhance the release of endorphins in both the brain and spinal cord. In contrast, high-frequency TEAS (100 Hz) stimulation promotes the release of dynorphins from the spinal cord [68]. A density-alternating wave (2 Hz/100 Hz) triggers the simultaneous release of endorphins, enkephalins, and dynorphins, leading to a synergistic analgesic effect [69]. However, use of TEAS should be prohibited in pregnant women and patients with pacemakers [70]. The results of this meta-analysis indicated that acupuncture did not demonstrate a significant advantage among the four physical intervention measures at 24 h after treatment. However, after 48 h of orthodontic treatment, acupuncture is more likely to be the best intervention measure with an absolute advantage. This could be because low-frequency TEAS takes longer to stimulate the production of endorphins [71], and thus takes longer to alleviate pain induced by orthodontic treatment. Nonetheless, low-frequency TEAS provides better and longer-lasting analgesic effects than does low-frequency TEAS.

Additionally, studies have shown that chewing gum can improve blood flow in the periodontal ligament, decrease the build-up of inflammatory substances that transmit pain signals, and ultimately decrease the perception of periodontal pain [72]. However, the effectiveness of chewing therapy as a means of pain control after orthodontic treatment remains debated [73]. Although our meta-analysis yielded positive results, the limited number of included studies and high levels of heterogeneity suggest the need for high-quality randomized controlled trials to confirm these findings.

## Conclusion

This meta-analysis demonstrated that LLLT, vibration, acupuncture, and chewing can alleviate the pain associated with orthodontic treatment. LLLT and vibration therapy not only have significant advantages in alleviating pain after orthodontic treatment but also have the effect of accelerating tooth movement, which has good clinical application prospects. However, further research is needed to determine how to balance pain relief and tooth movement acceleration to achieve optimal results. Acupuncture has been shown to have an absolute advantage in providing long-lasting and stable pain relief 48 h after orthodontic treatment. TEAS, an innovative approach in traditional Chinese medicine, has demonstrated excellent pain relief after orthodontic treatment. Further research is required to determine the most suitable equipment parameters for acupuncture.

## Supporting information

S1 Table The PRISMA network meta-analysis checklist. (DOCX)

S1 Fig Effect of low-level laser therapy (LLLT) on pain control at 24 h after orthodontic treatment. (TIF)

S2 Fig Effect of low-level laser therapy (LLLT) on pain control at 48 h after orthodontic treatment. (TIF)

S3 Fig Effect of vibration on pain control at 24 h after orthodontic treatment. (TIF)

S4 Fig Effect of vibration on pain control at 48 h after orthodontic treatment. (TIF)

S5 Fig Effect of chewing on pain control at 24 h after orthodontic treatment. (TIF)

S6 Fig Effect of chewing on pain control at 48 h after orthodontic treatment. (TIF)

S7 Fig Effect of acupuncture on pain control at 24 h after orthodontic treatment. (TIF)

S8 Fig Effect of acupuncture on pain control at 48 h after orthodontic treatment. (TIF)
