# Supplementary figures and images for: The effect of physical interventions on pain control after orthodontic treatment: A systematic review and network meta-analysis
Source: PLoS One. 2024 Feb 22;19(2):e0297783. doi: 10.1371/journal.pone.0297783 (PMC10883545; doi:10.1371/journal.pone.0297783)

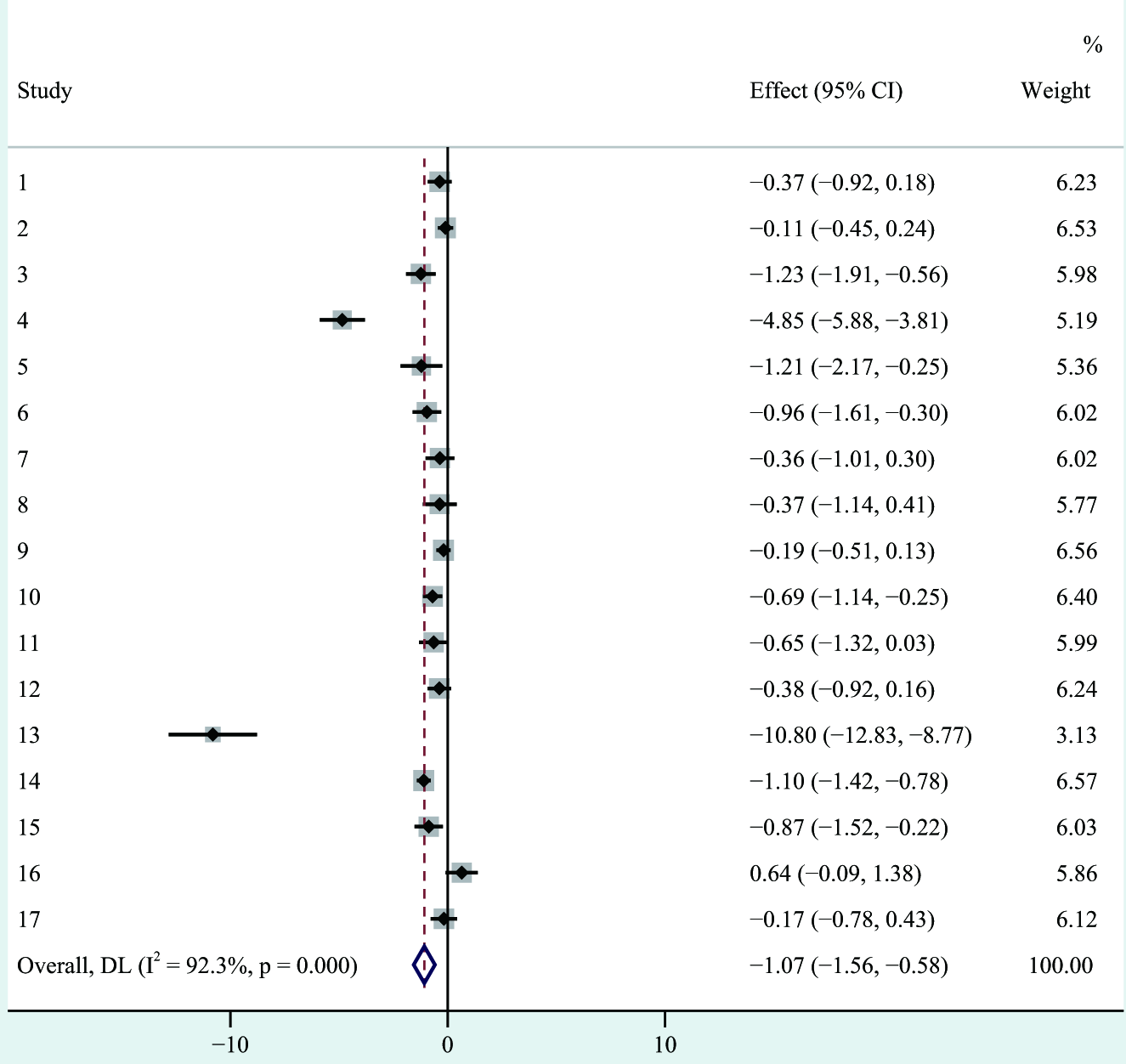

Supplement: S1 Fig — (TIF) [file pone.0297783.s002.tif]

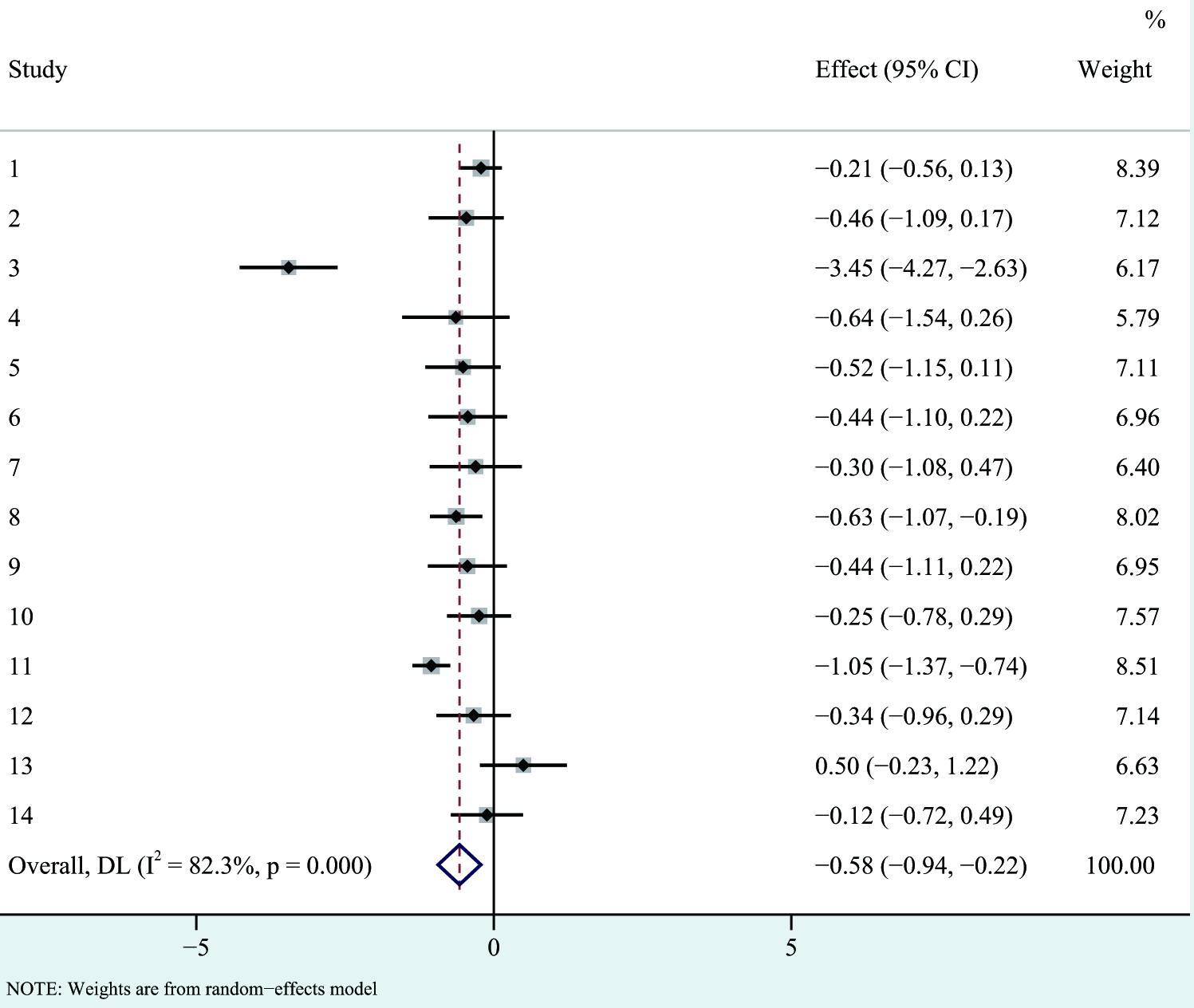

Supplement: S2 Fig — (TIF) [file pone.0297783.s003.tif]

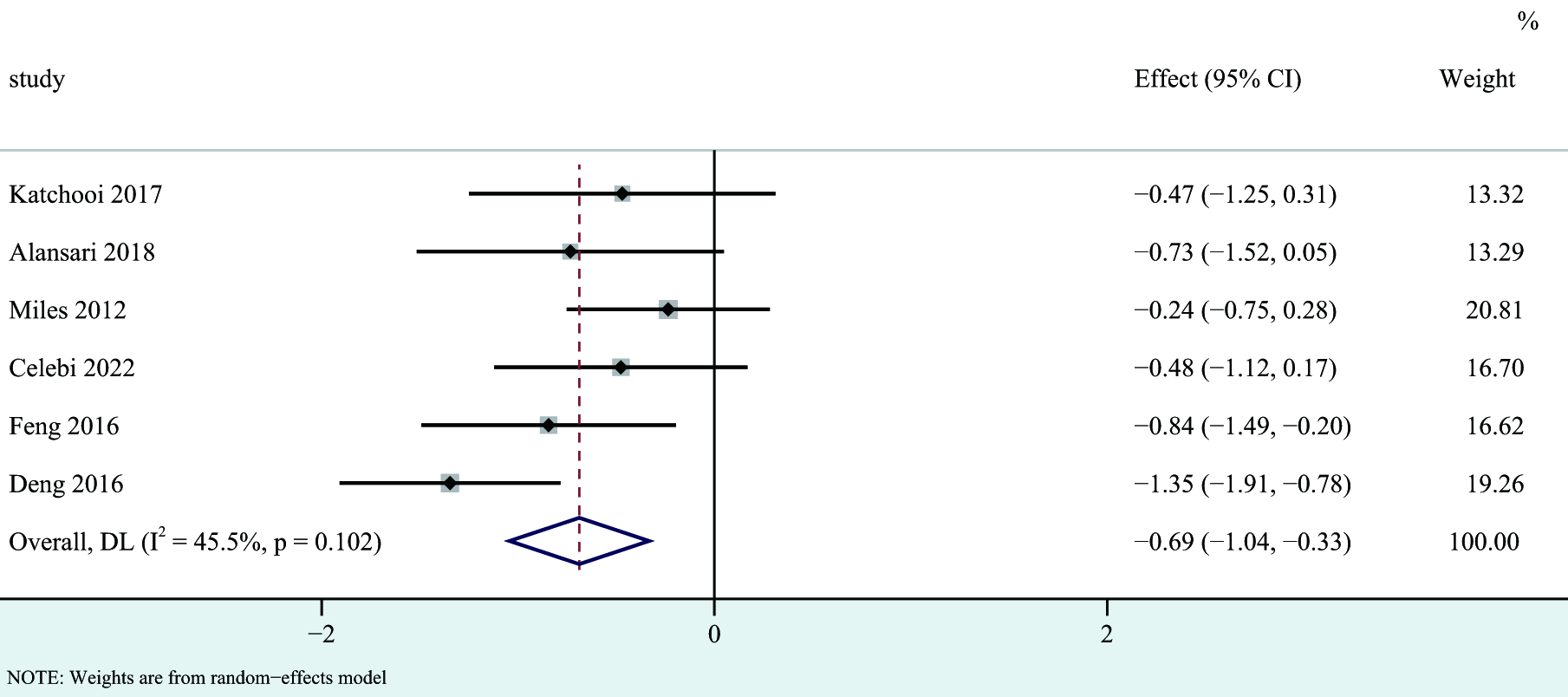

Supplement: S3 Fig — (TIF) [file pone.0297783.s004.tif]

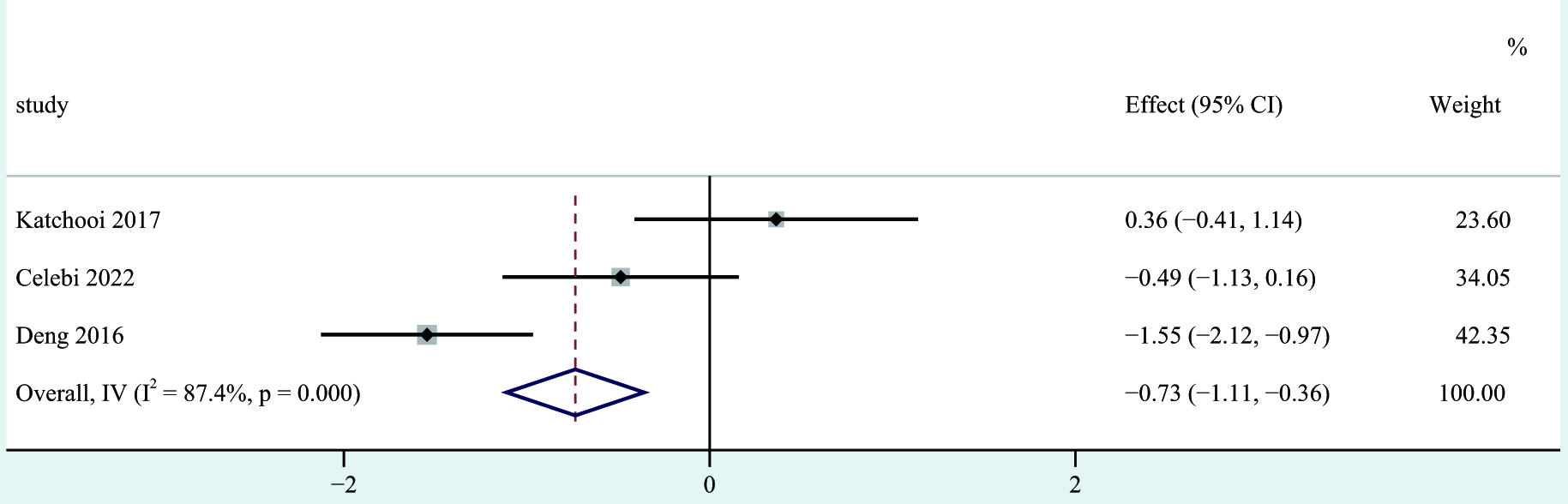

Supplement: S4 Fig — (TIF) [file pone.0297783.s005.tif]

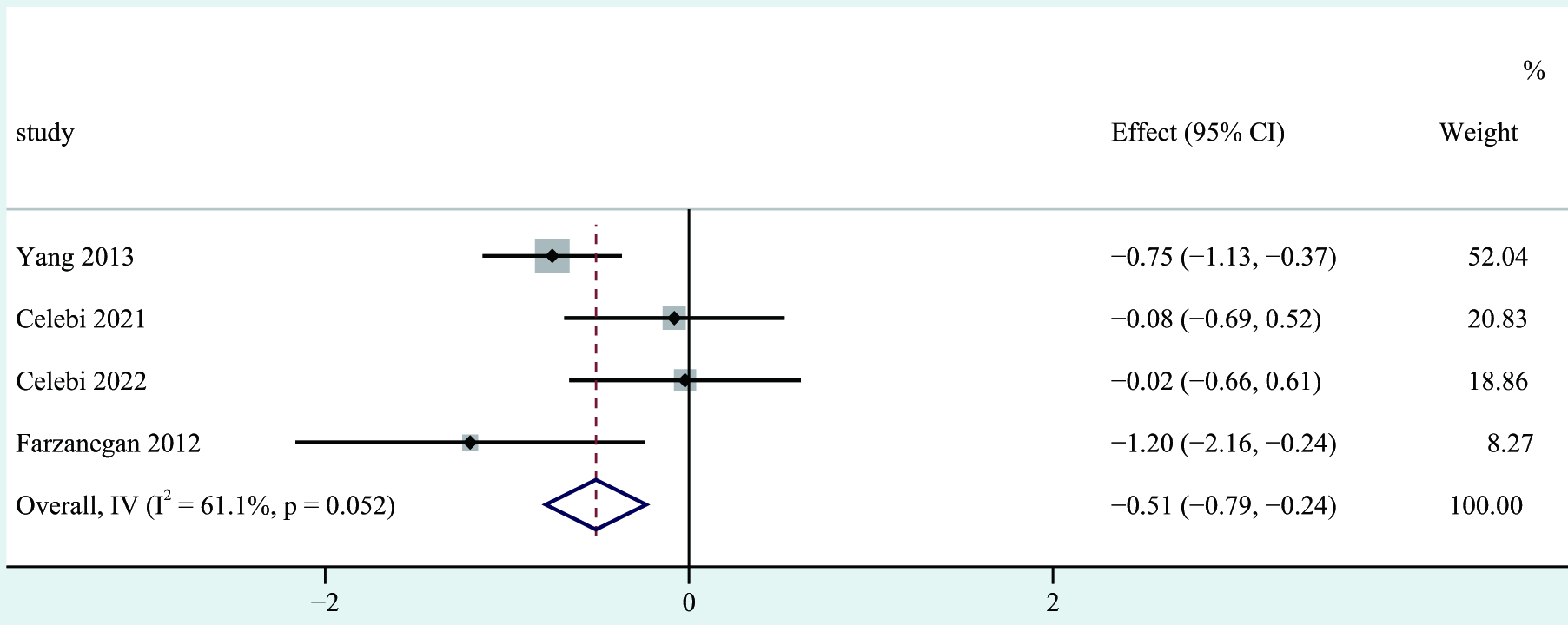

Supplement: S5 Fig — (TIF) [file pone.0297783.s006.tif]

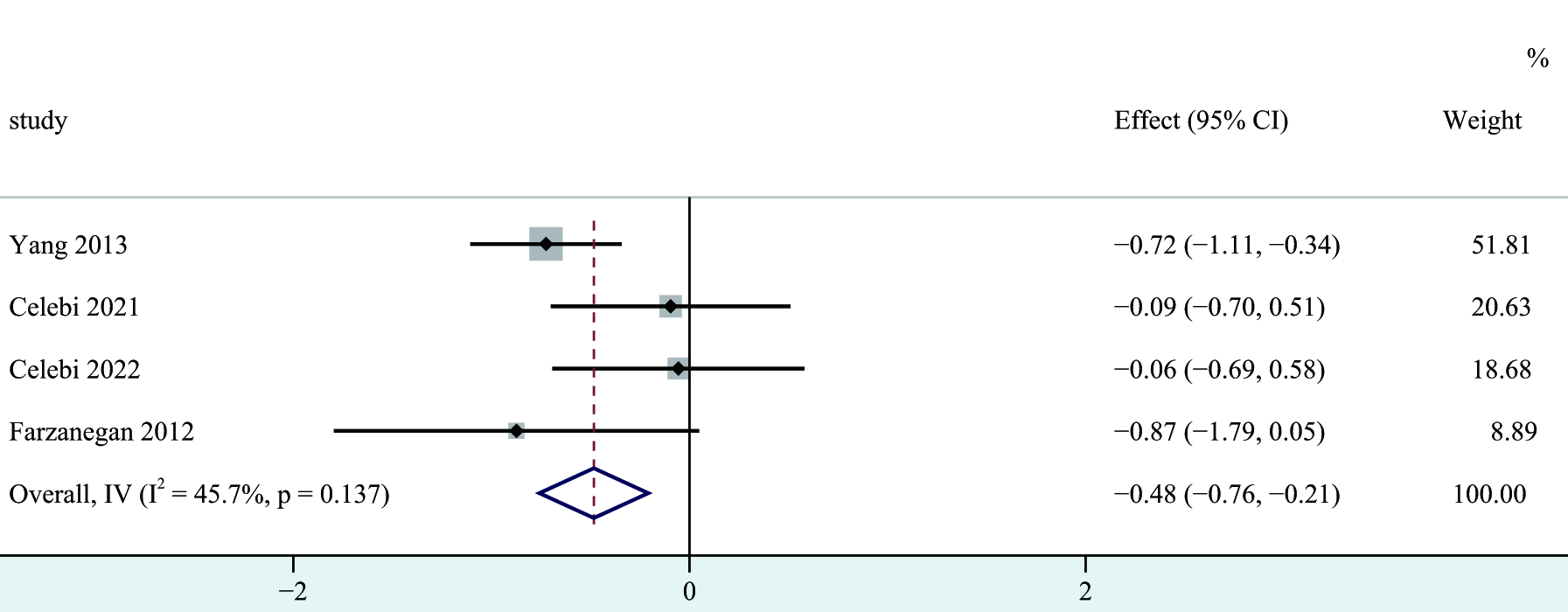

Supplement: S6 Fig — (TIF) [file pone.0297783.s007.tif]

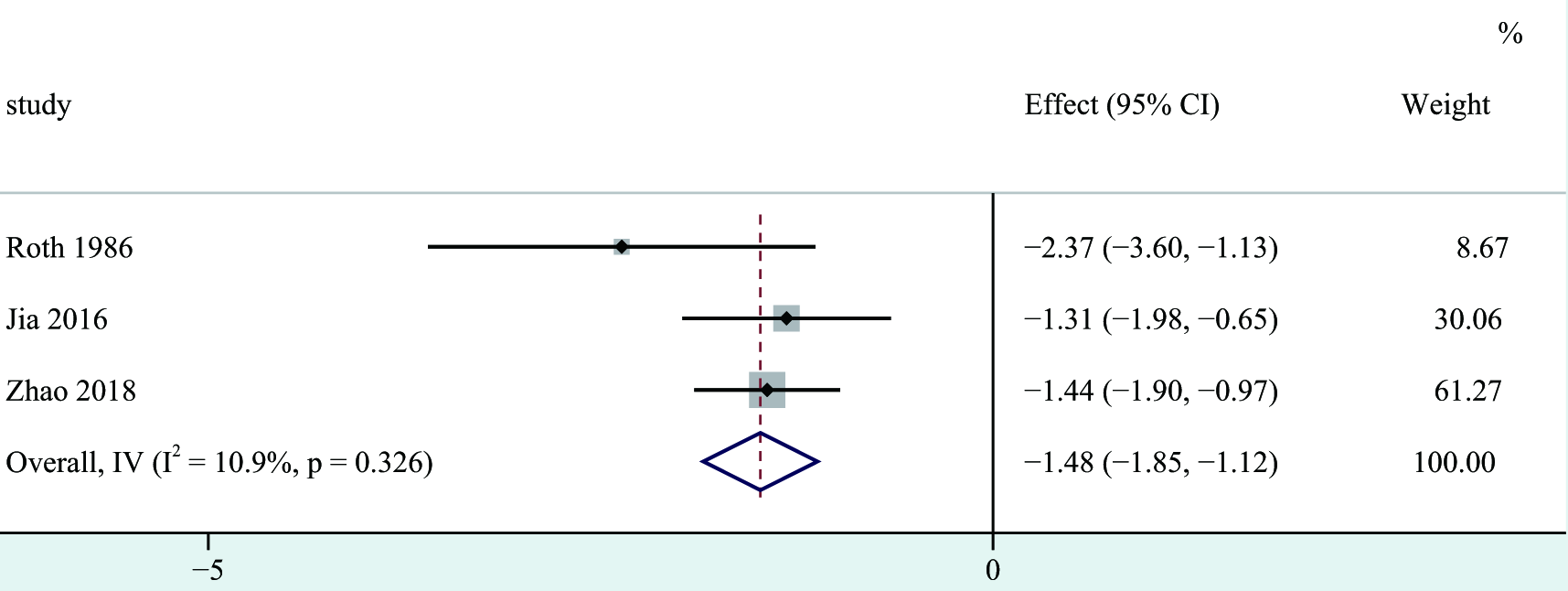

Supplement: S7 Fig — (TIF) [file pone.0297783.s008.tif]

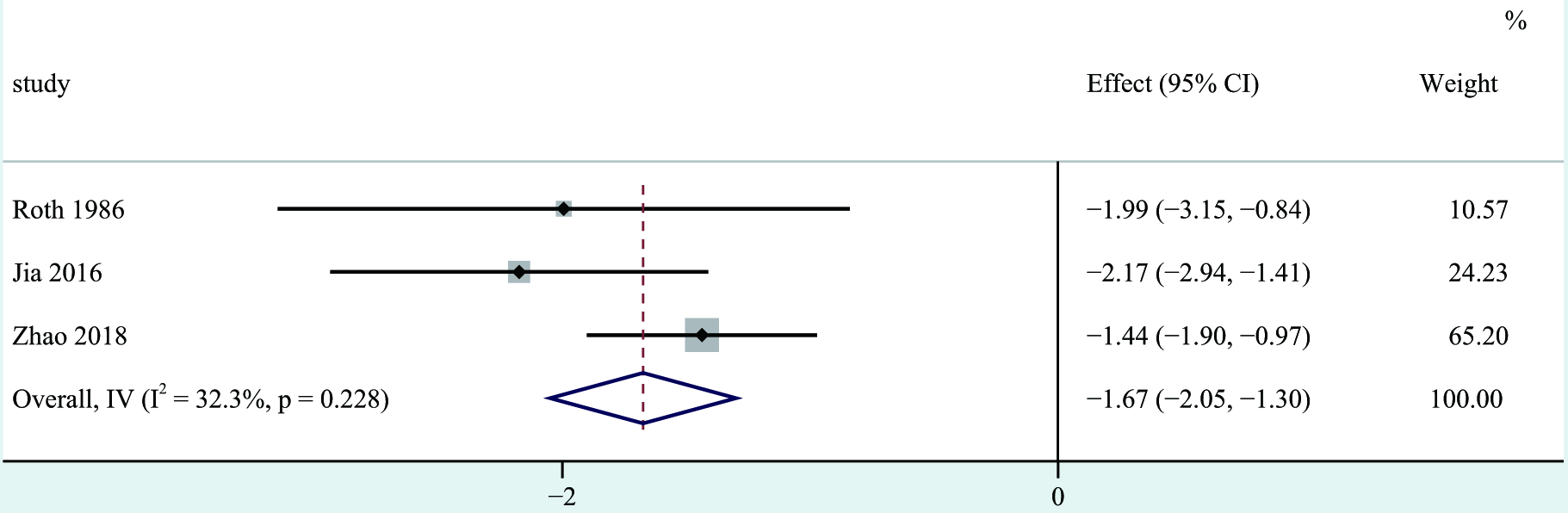

Supplement: S8 Fig — (TIF) [file pone.0297783.s009.tif]
